# Synchronous triple carcinoma of the colon and rectum

**DOI:** 10.1186/1477-7819-11-66

**Published:** 2013-03-13

**Authors:** Chien-Chih Yeh, Sheng-Chuan Hsi, Chih-Pin Chuu, Yung-Hsi Kao

**Affiliations:** 1 Division of Colon and Rectal Surgery, Taoyuan Armed Forces General Hospital, Taoyuan, Taiwan; 2 Department of Surgery, Taoyuan Armed Forces General Hospital, Chung-Shin Road, Lungtan, Taoyuan, Taiwan; 3 Institute of Cellular and System Medicine, National Health Research Institutes, Keyan Road, Chunan, Miaoli, Taiwan; 4 Department of Life Sciences, National Central University, Jhongda Road, Jhongli, Taoyuan, Taiwan

**Keywords:** Synchronous colorectal cancer, Colon, Rectum, Taiwan

## Abstract

Synchronous multiple colorectal cancers are defined as multiple malignant colorectal tumors that occur simultaneously. All tumors are distant from each other, and none are the result of metastasis from other tumors. Here, we present a case of a 79-year-old man who was admitted to our hospital because of a 3-month history of abdominal pain associated with anemia, loss of appetite, and body weight loss. The patient did not have a family history of cancer. Computed tomography revealed bowel wall thickness and mesentery inflammation at the hepatic flexure of the colon and cecum. Colonoscopy revealed a tumor located 10 cm from the anal verge. Colonoscopic examination of the large bowel was not possible because of bowel obstruction due to the rectal tumor. Synchronous triple adenocarcinoma of the colon and rectum was confirmed by pathologic examination. The tumor was surgically resected by two-segment resection of the colon, low anterior resection, and right hemicolectomy. We used intraoperative colonoscopy to confirm that there were no other lesions after the resection of the three tumors. To the best of our knowledge, this is the first case of synchronous triple carcinoma of the colon and rectum in Taiwan. We consider that comprehensive preoperative study, extensive intraoperative exploration, and radical resection can increase the survival rate of patients with synchronous multiple colorectal cancers.

## Background

The colon is the organ most frequently involved in synchronous malignancy, especially in patients of advanced age [1-3]. Synchronous cancers are exceptional and the frequency of multiple synchronous colon and rectal carcinoma is even rarer, particularly in the Taiwanese population. We report a case of synchronous triple carcinoma arising from the colon and the rectum in an elderly Taiwanese man without a family history of colon cancer.

## Case presentation

**Figure 1:**
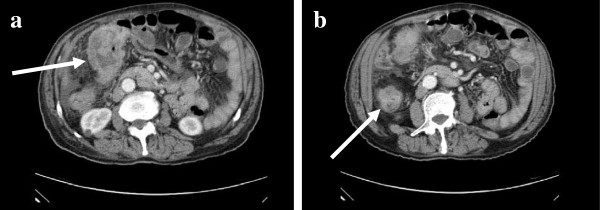
**Abdominal computed tomography (CT) suggested a large tumor of hepatic flexure of the colon (a) (arrow).** Another tumor of the cecum is visible on a different cut (**b**) (arrow).

**Figure 2:**
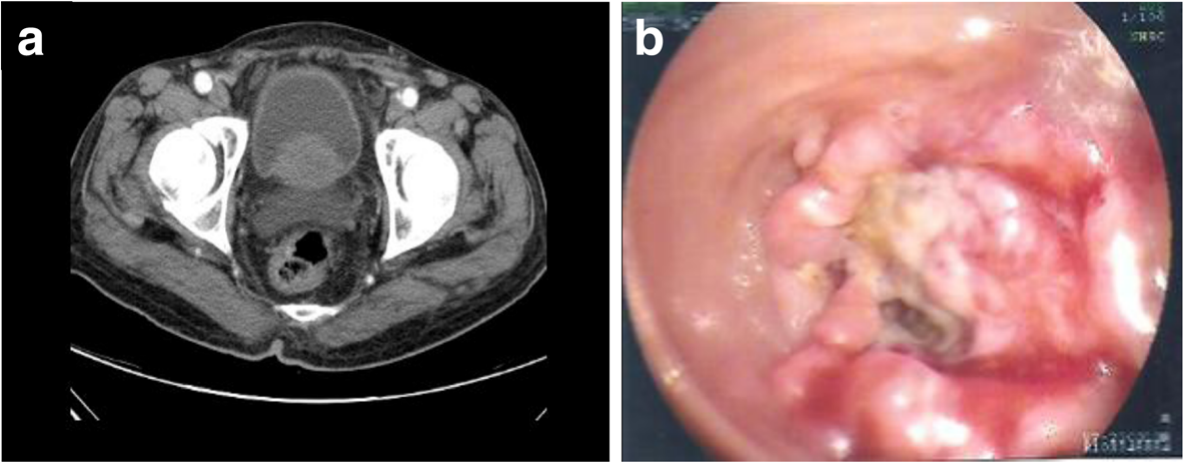
**Abdominal computed tomography (CT) shows a tumor-like lesion in the rectal region (a).** Colonoscopy shows one annular tumor located about 10 cm from the anal verge (**b**). Note that the lumen is almost completely obstructed.

A 79-year-old man with a history of hypertension presented with a 3-month history of abdominal pain associated with hematochezia, poor appetite, and body weight loss. The patient did not have a family history of cancer. Physical examination revealed tenderness at the right upper quadrant of the abdomen. Laboratory studies revealed a hematocrit value of 24.9%, a hemaglobin level of 7.5 g/dl, and a CA-199 value of 486.2 U/ml. Other cancer markers such as á-FP, CEA, and PSA were within the normal range. No anomalies were detected on chest X-ray, abdominal sonography and fiber-optic gastroscopic examination. Computed tomography (CT) of the abdomen revealed obvious bowel wall thickness and mesentery inflammation in the hepatic flexure of the colon and cecum (Figure 1). Rectal tumor was suspected. The colonoscopic examination revealed a tumor located 10 cm from the anal verge. Examination of the entire colon was not possible because of bowel obstruction due to the rectal tumor (Figure 2b). Histopathologic examination of the biopsy specimen showed moderately differentiated adenocarcinoma. Exploratory laparotomy revealed a tumor with a diameter of 3 cm in the rectal region. A second tumor with a diameter of 7 cm was also found at the hepatic flexure of the colon. A third tumor with a diameter of 4 cm was found in the cecum. No metastatic tumors were detected in the abdominal cavity or liver. Enlarged lymph nodes were present in the mesentery near the hepatic flexure of the colon. Low anterior resection and an extended right hemicolectomy were performed. Histopathologic examination of the removed specimens revealed three macroscopically distinct tumors. Two distinct tumors were found, one at the right side approximately 3 cm from the ileocecal valve and the other approximately 25 cm from the ileocecal valve. Microscopic examination of the specimens revealed that the adenocarcinoma was moderately differentiated and a metastatic tumor was found in the regional lymph nodes of the hepatic flexure of the colon. (stage IIIc, T3N2M0 according to the American Joint Committee on Cancer tumor-node-metastasis system) The sample collection was approved by the Ethics Committee.

The incidence of multiple primary cancers in the colon and rectum is about 2 to 5% [4-9]. The actual incidence is probably higher because not every case is identified. This incidence increases in patients with a history of adenomatosis polyposis, hereditary non-polyposis colorectal cancer, or ulcerative colitis [10,11]. Synchronous colorectal cancers are defined as tumors diagnosed either preoperatively, during an operation by palpation, or postoperatively by colonoscopy within a period of less than 6 months. Synchronous tumors are normally at least 4 cm from each other with no intrusion into the submucosal layer of the colon [4]. Herein, we report a rare case of triple synchronous cancers arising from the colon and the rectum in an elderly patient without a family history of cancer or genetic predisposing factors.

The preoperative diagnosis of multiple synchronous colorectal carcinomas remains difficult. Additional tumors may be ignored or missed after discovery of the first tumor. In this case, we focused on the patient’s hepatic flexure of the colon and cecum according to clinical symptoms, signs, and image study findings. The rectal cancer was unexpectedly identified by colonoscopy as this small annular tumor was not obvious on CT (Figure 2a). It is recommended that routine preoperative colonoscopy should be done in patients with colorectal cancer in order to identify synchronous polyps or cancers. These polyp lesions or tumors can be removed endoscopically or by surgery. Otherwise, the polyps or tumors may remain undetected at the time of the surgery [12]. Preoperative colonoscopy of the entire colon is often unobtainable due to bowel obstruction by the tumor, poor bowel preparation, or technical limitations. In this case, intraoperative colonoscopy is a possible alternative examination [13]. After surgical resection of the triple colon cancers, we used intraoperative colonoscopy to confirm that there were no other tumors. However, not every investigator agrees on the effectiveness of intraoperative colonoscopy. Some studies reported that the incidence of cancer outside the surgical location is high and that intraoperative colonoscopy increases the risk of infection [14].

Precise detection of all synchronous colorectal cancers before operation is very important, as the number and location of tumors may affect the surgical procedure. Re-operation is required for non-detected synchronous cancers that usually ultimately present as early metachronous carcinoma of advanced stage. As the occluded distal lesion may cause the proximal lesions difficult to be found by barium enema or colonoscopy, it makes synchronous colorectal cancers hard to be diagnosed. Therefore, it is necessary to carefully examine the entire large bowel both pre- and intra-operatively in colon cancer patients. It is also important to palpate the entire colon as well as check pathological specimens thoroughly during the operation. These procedures can help in minimizing misdiagnosis of synchronous cancers.

CT colonography and magnetic resonance (MR) colonography are two mini-invasive imaging methods that were described in 1994 and 1997, respectively, and were used to examine the entire colon on the basis of the respective CT and MR data sets [15,16]. The major advantages of these two imaging methods are that they can help visualize the entire colorectum in patients with incomplete colonoscopy. Potential synchronous colon lesions may be detected using these approaches [17-23]. A recent study indicated that whole-body Positron emission tomography (PET)/CT colonography plays an important role as a whole-body staging modality in patients with colorectal cancer, especially those patients with bowel obstructive condition or questionable synchronous lesions. It also provides additional evaluation of the colon proximal to the stenosis [24-26]. These examination methods of the bowel are attractive alternatives for the use of whole colorectal lesions detection. Moreover, this valuable information enables the surgeon to decide the optimum surgery for the patient. These techniques could further produce more images of the entire colon and extracolonic abnormalities than the traditional methods. Unfortunately, these facilities are presently not widespread available in general hospitals, and there are still considerable variations in diagnostic results depending on the interpretation of institutional personnel.

Operative techniques for managing multiple lesions or cancers need to be tailored to the individual, based on the location, status of invasion, and patient’s health condition. The initial operative procedure for multiple colorectal carcinomas is controversial. Some studies have suggested total or subtotal colectomy to remove any potential existing synchronous tumors or polyps that have not been detected [27-29]. It is thought that the removal of the entire colon will prevent development of metachronous tumors. However, other studies recommend a more conservative surgical approach [30,31]. We performed surgery according to the latter recommendation with two-segment resection of the colon, low anterior resection and right hemicolectomy. However, we used intraoperative colonoscopy to confirm that there were no other lesions after the operation. It should be noted that there is no significant difference in survival between multiple and single colorectal cancers [32].

## Conclusions

To the best of our knowledge, the present report is the first case of synchronous multiple colorectal cancers in Taiwan. The patient had no family history of colon cancer. The first tumor measuring 3 cm in diameter was in the rectal region, the second tumor measuring 7 cm in diameter was in the hepatic flexure of the colon, and the third tumor measuring 4 cm in diameter was in the cecum. For treatment of synchronous multiple colorectal cancers, identification of concurrent lesions is vital to improve prognosis. Therefore, a thorough evaluation of the entire colon is very important. Treatment of synchronous colorectal cancers is not different from that of single colorectal cancers. Comprehensive preoperative study, extensive intraoperative exploration, and radical resection allow for early diagnosis and treatment of synchronous multiple colorectal cancers, which can increase the survival rate of patients with these cancers.

## Consent

Written informed consent was obtained from the patient for publication of this case report and any accompanying images. A copy of the written consent is available for review by the Editor-in-Chief of this journal.

## Abbreviations

CT: computed tomography; MR: magnetic resonance; PET: positron emission tomography.

## Competing interests

The authors declare that they have no competing interests.

## Authors’ contributions

C-CY wrote the main manuscript and performed the operation. S-CH, C-PC, and Y-HK revised the manuscript for important intellectual content, and provided the final approval for the version to be submitted for publication. All authors read and approved the final manuscript.
